# Development of New 1,3-Diazaphenoxazine Derivatives (ThioG-Grasp) to Covalently Capture 8-Thioguanosine

**DOI:** 10.3390/molecules20011078

**Published:** 2015-01-09

**Authors:** Yasufumi Fuchi, Hideto Obayashi, Shigeki Sasaki

**Affiliations:** Graduate School of Pharmaceutical Sciences, Kyushu University, 3-1-1 Maidashi, Higashi-ku, Fukuoka 812-8582, Japan; E-Mails: fuchi@phar.kyushu-u.ac.jp (Y.F.); 2PS14001P@s.kyushu-u.ac.jp (H.O.)

**Keywords:** oxidative damage, oxidized nucleoside, 8-oxoguanosine, 8-nitroguanosine, 8-thioguanosine

## Abstract

The derivatives of 8-thioguanosine are thought to be included in the signal transduction system related to 8-nitroguanosine. In this study, we attempted to develop new 1,3-diazaphenoxazine (G-clamp) derivatives to covalently capture 8-thioguanosine (thioG-grasp). It was expected that the chlorine atom at the end of the linker would be displaced by the nucleophilic attack by the sulfur atom of 8-thioguanosine via multiple hydrogen-bonded complexes. The thioG-grasp derivative with a propyl linker reacted efficiently with 8-thioguanosine to form the corresponding adduct.

## 1. Introduction

Reactive oxygen species (ROS) and reactive nitrogen oxide species (RNOS) are the chemical sources of oxidative stress, and they oxidize nucleic acids to produce the 8-oxoguanosine and 8-nitroguanosine derivatives [1,2,3]. The oxidized nucleosides/nucleotides are highly mutagenic and are regarded as biomarkers of oxidative stress [4]. On the other hand, recent studies have revealed that nitrated-guanosine derivatives also play important roles as signal messengers; 8-nitroguanosine-3′,5′-cyclic monophosphate (8-nitro-cGMP) is generated from guanosine- 5′-triphosphate (GTP) in response to the production of peroxynitrite (ONOO^−^) and reacts with the sulfhydryl groups of proteins [5], H_2_S/HS^−^ [6] or persulfide [7] to form 8-adduct-cGMP (S-guanylation) or 8-thio-cGMP. These metabolic cycles have led to the proposal of a new signaling pathway mediated by 8-nitro-cGMP. 8-Thio-cGMP may be returned to cGMP by ROS such as hydrogen peroxide in cells, but its biochemical role is not well understood. Thus, selective molecules that can form a covalently bonded complex with the 8-thioG derivative are required to further understand their biological functions. However, there is no specific recognition compound for 8-thioguanosine derivatives. In this study, we reported new 1,3-diazaphenoxazine nucleoside derivatives (thioG-grasp) that exhibit an efficient covalent capture of 8-thioguanosine via the formation of multiple hydrogen-bonded complexes.

**Figure 1:**
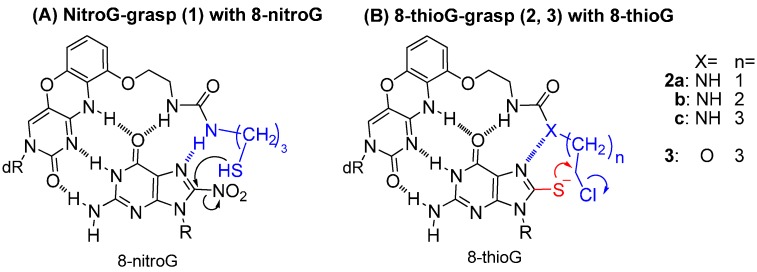
Molecular design of the selective capture molecules for 8-nitroG and 8-thioG. dR and R represent 3′,5′-diOTBS-2′-deoxyribosyl and triacetyl ribosyl groups, respectively.

We have focused on the development of recognition molecules for the 8-oxidized guanosine derivative based on the tricyclic cytosine analog “G-clamp” [8,9,10,11,12], and have reported the “8-oxoG-clamp” derivatives for the selective fluorescent detection of 8-oxo-dG [13,14,15]. Most recently, a new 1,3-diazaphenoxazine nucleoside derivative bearing a thiol arm, nitroG-grasp (**1**), has demonstrated the efficient capture of 8-nitroguanosine via multiple hydrogen-bonded complexes [16]. The displacement reactivity of nitroG-grasp (**1**) depends on the thiol p*K*_a_ and the length of the alkyl linker between the urea and the thiol group. For the new capture molecules for 8-thioguanosine, we based the design on a hydrogen bonded complex, such as that between the 8-nitroguanine portion and the 1,3-diazaphenoxazine portion connecting the urea-linker (Figure 1A). A nucleophilic attack of the 8-sulfur atom on the chloride leaving group was expected to form the corresponding covalent bond. The urea-type linker (X=NH) and the carbamate-type linker (X=O) were anticipated to form a suitable hydrogen bond with the thioenolate form or with the thioamide form, respectively (Figure 1B, nitroG-grasp, **2a–c**, **3**). In this study, we report the synthesis of 8-thioG-grasp derivatives and evaluated their reactivity with 8-thioguanosine.

## 2. Results and Discussion

### 2.1. Chemistry

**Scheme 1:**
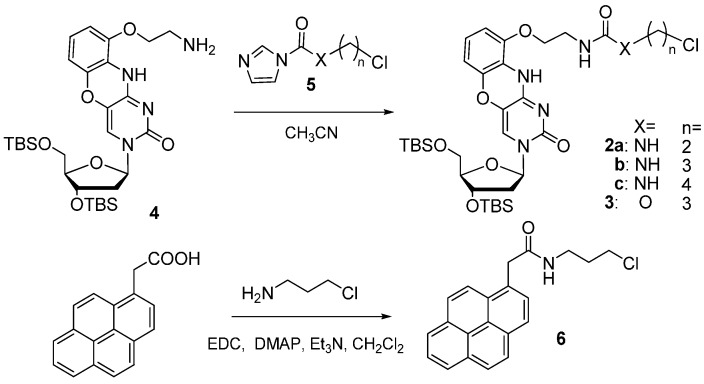
The synthesis of the 8-thioG-grasp derivatives.

In this study, 2′,3′,5′-tri-*O*-acetyl-8-thioguanosine (tri-Ac-8-thioG) was used as a substrate and was synthesized from 2′,3′,5′-tri-O-acetyl-8-bromoguanosine according to the literature [17]. The 8-thioG-grasp derivatives were synthesized through the reaction between the imidazole linker unit **5** and the amino group of the 3′,5′-O-diTBDMS-G-clamp unit **4** as a common intermediate (Scheme 1). The chloroalkylamine or chloropropanol were treated with carbonylimidazole in CH_3_CN in the presence of triethylamine to form the corresponding imidazole intermediates **5**, which reacted with **4** to produce the desired 8-thioG-grasp derivatives. *N*-(3-Chloropropyl)-2-(pyrene-1-yl) acetamide **6** was synthesized from 1-pyrene-acetic acid as a control compound with no complexation site for 8-thioG.

### 2.2. Reaction of 8-ThioG-Grasp with 8-ThioG

**Figure 2:**
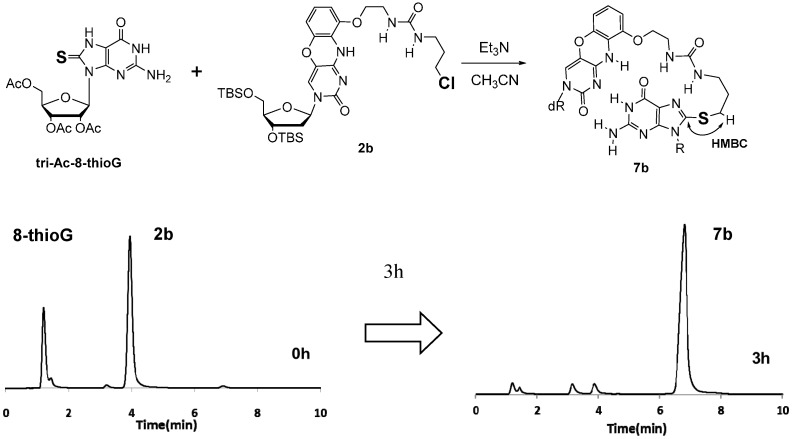
Reaction of 8-thioG-grasp with tri-Ac-8-thioG monitored by HPLC. dR and R represent 3′,5′-diOTBS-2′-deoxyribosyl and triacetyl ribosyl groups, respectively. The reaction was performed using 0.4 mM each of **2b** and 8-thioG in the presence of 20 mM Et_3_N in CH_3_CN at 50 °C. HPLC conditions: column: Xbridge C8 3.5 μm, 3.0 mm × 100 mm; solvents: (**A**) 0.1 M TEAA buffer at pH 7.0 and (**B**) CH_3_CN, A/B = 20:80; flow rate: 0.5 mL/min; monitored by UV at 254 nm.

The reaction of the 8-thioG-grasp derivatives with triAc-8-thioG was performed at 50 °C in CH_3_CN in the presence of Et_3_N, and the reaction progress was monitored by HPLC. An equimolar mixture of 8-thioG-grasp (**2b**) and triAc-8-thioG formed an adduct in a nearly quantitative yield as a single product within 3 h (Figure 2).

This product was isolated, and its structure was confirmed as depicted in **7b** by ^1^H-NMR, ESI-MS, and HMBC (see the Supporting Information). The HMBC spectrum indicated the distinct correlation between the C8 of the 8-thioguanine unit and the methylene protons next to the sulfur atom of **7b**.

### 2.3. Comparison of the Reactivity between 8-ThioG-Grasp and the Control Compound

**Figure 3:**
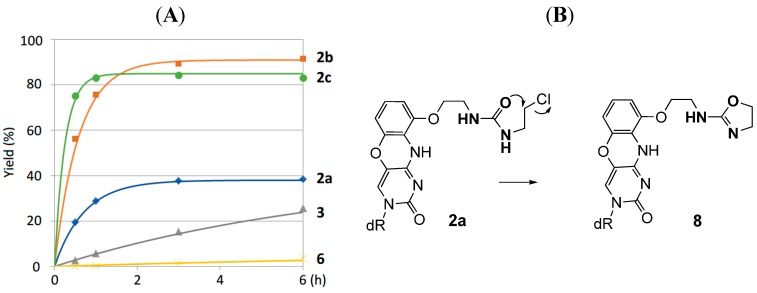
(**A**) Comparison of the time courses of the reactions with 8-thioG-grasp (**2a–c** and **3**) and the control compound **6**. (**B**) Byproduct **8** formed via the intramolecular reaction of **2a**. Product yields were obtained at the indicated time points by HPLC analysis, as described in the footnote to Figure 2. dR represents the 3′,5′-diOTBS-2′-deoxyribosyl group.

The time courses of the reaction between the 8-thioG-grasp derivatives **2a–c**, **3** and triAc-8-thioG are compared in Figure 3. **2b** and **2c** exhibited efficient reactivity, and a relatively slow reaction was observed with **2a**. In the reaction of **2a**, the formation of the amino-oxazoline ring (**8**) as a byproduct decreased the adduct yield (Figure 3).

**Figure 4:**
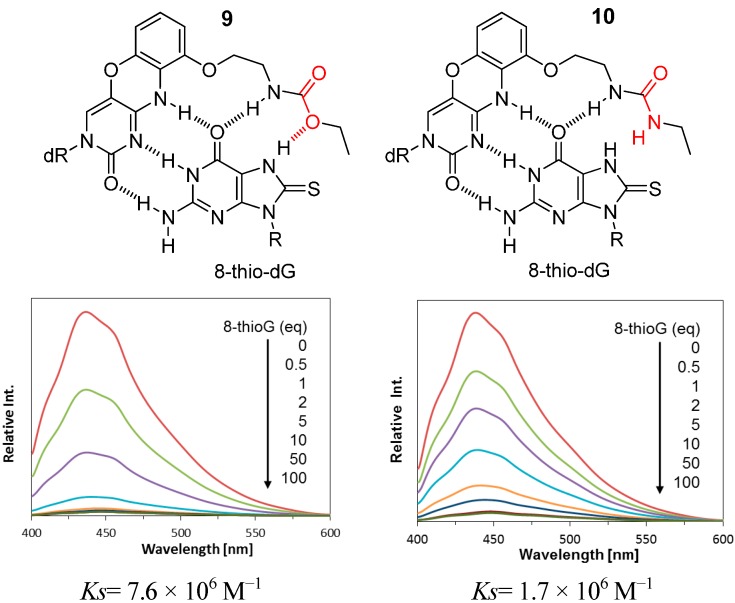
Proposed complexation of 8-thioG with the carbamate or the urea type. dR and R represent 3′, 5′-diOTBS-2′-deoxyribosyl and triacetyl ribosyl groups, respectively.

In contrast, the carbamate type **3** showed a significantly decreased efficiency compared with the compounds **2** with the urea-type linker. This is of great interest because 8-thioketo tautomer of 8-thioG is stable in neutral organic solvents [18] and forms more stable complexes with the carbamate type **9** than with the urea type **10** (*K_s_* in CHCl_3_, **9**: 7.6 × 10^6^ M^−1^ *vs.* **10**: 1.7 × 10^6^ M^−1^) (Figure 4). Accordingly, it is reasonably explained that the 7N-H of 8-thioG is deprotonated by Et_3_N to form the 8-thioenolate, thereby facilitating hydrogen-bonded complexes with the urea-type compounds (**2b** and **2c**) such as shown in Figure 1B to exhibit efficient reactivity. As the p*K*_a_ value of 7N-H of 8-thioguanosine is around 8.5 (see the Supporting Information), Et_3_N is a suitable base for its deprotonation. No adduct was formed with *N*-(3-chloropropyl)-2-(pyrene-1-yl) acetamide (**6**), a control without a binding site, emphasizing the contribution of the hydrogen-bonded complexation of **2b** and **2c** for efficient reactivity. Among the urea-type 8-thioG-grasp derivatives, **2a** produced the adduct in a low yield. The HPLC monitoring showed that **2a** formed the byproduct **8** as a major peak, which structure was determined by H-NMR, ESI-MS and 2D-HMBC, as shown in Figure 3. The amino-oxazoline was formed by intramolecular displacement, which was faster than the intermolecular nucleophilic attack from 8-thioG. It should be emphasized that the displacement of the sulfur atom of 8-thioG in the complex with **2b** or **2c** is favorable compared with the intramolecular amino-oxazoline ring formation because this type of amino-oxazoline ring formation was not observed for **2b** or **2c**.

## 3. Experimental Section

### 3.1. General Information

The reagents and solvents were purchased from commercial suppliers and were used without purification. The ^1^H- and ^13^C- NMR spectra were recorded on a Bruker Avance III spectrometer. The 2D-NMR spectra were measured on a Varian Inova 500 instrument. The IR spectra were recorded on a Perkin Elmer Spectrum One FT-IR spectrometer. The ESI-HRMS spectra were measured on an Applied Biosystems Mariner Biospectrometry Workstation using neurotensin, angiotensin I, bradykinin and picolinic acid as the internal standards.

### 3.2. Chemistry

#### 3.2.1. General Synthesis of 8-ThioG-Grasp Derivatives

1,1′-Carbonyldiimidazole (5 equiv.) was added to a solution of chloroalkylamine hydrochloride (5 equiv.) in anhydrous CH_3_CN (0.05 M in G-clamp unit) under an argon atmosphere. The reaction mixture was stirred at room temperature for 30 min, followed by the addition of the G-clamp unit **4** (1 equiv.) and Et_3_N (8 equiv.). After stirring overnight at room temperature, saturated aqueous NaHCO_3_ solution was added to the reaction mixture, which was extracted with CHCl_3_. The organic layer was washed with brine, dried over Na_2_SO_4_ and evaporated *in vacuo*. The resulting residue was purified by silica gel column chromatography (CH_2_Cl_2_/MeOH =100:0 to 95:5) to give **2a–c** and **3** as light yellow foams.

*1-(2-((3-((2R,4S,5R)-4-((tert-Butyldimethylsilyl)oxy)-5-(((tert-butyldimethylsilyl)oxy)methyl)-tetrahydrofuran-2-yl)-2-oxo-2,10-dihydro-3H-benzo[b]pyrimido[4,5-e][1,4]oxazin-9-yl)oxy)ethyl)-3-(2-chloroethyl)urea*. (C2-8-ThioG-grasp, **2a**): **2a** was synthesized using G-clamp unit **4** and 2-chloroethylamine in 48% as a light yellow foam. ^1^H-NMR (400 MHz, CD_3_OD) δ (ppm) 7.62 (1H, s), 6.82 (1H, t, *J* = 8.6 Hz), 6.60 (1H, d, *J* = 8.6 Hz), 6.35 (1H, d, *J* = 8.6 Hz), 6.17 (1H, t, *J* = 6.4 Hz), 4.46 (1H, dt, *J* = 6.4, 3.4 Hz), 4.05 (2H, t, *J* = 4.9 Hz), 3.96–3.93 (2H, m), 3.82 (1H, dd, *J* = 12.1, 3.4 Hz), 3.57 (2H, t, *J* = 6.0 Hz), 3.54 (2H, t, *J* = 5.2 Hz), 3.45 (2H, t, *J* = 6.4 Hz), 2.33 (1H, ddd, *J* = 13.4, 6.1, 4.6 Hz), 2.11 (1H, dt, *J* = 13.4, 6.4 Hz), 0.97 (9H, s), 0.91 (9H, s,), 0.18 (3H, s), 0.16 (3H, s), 0.11 (6H, s). ^13^C-NMR (125 MHz, CDCl_3_) δ (ppm) 158.53, 152.89, 146.88, 143.06, 127.52, 124.49, 122.97, 115.06, 108.21, 107.54, 87.75, 86.00, 70.84, 69.22, 62.32, 42.09, 41.89, 39.33, 29.28, 26.04, 25.71, 18.49, 17.94, −4.58, −4.90, −5.45, −5.52. IR (cm^−1^): 2929, 1673, 1557, 1473. HR ESI-MS (*m*/*z*): Calcd. for [C_32_H_53_ClN_5_O_7_Si_2_]^+^: 710.3167 ([M+H]^+^), found 710.3176.

*1-(2-((3-((2R,4S,5R)-4-((tert-Butyldimethylsilyl)oxy)-5-(((tert-butyldimethylsilyl)oxy)methyl)-tetrahydrofuran-2-yl)-2-oxo-2,10-dihydro-3H-benzo[b]pyrimido[4,5-e][1,4]oxazin-9-yl)oxy)ethyl)-3-(3-chloropropyl)urea*. (C3-8-ThioG-grasp, **2b**): **2b** was synthesized using G-clamp unit **4** and 3-chloropropylamine in 46% as a light yellow foam. ^1^H-NMR (400 MHz, CD_3_OD) δ (ppm) 7.62 (1H, s), 6.82 (1H, t, *J* = 8.2 Hz), 6.60 (1H, dd, *J* = 8.4, 1.2 Hz), 6.35 (1H, dd, *J* = 8.2, 1.2 Hz), 6.17 (1H, t, *J* = 6.4 Hz), 4.47 (1H, dt, *J* = 5.7, 3.7 Hz), 4.05 (2H, t, *J* = 5.2 Hz), 3.95 (1H, t, *J* = 3.1 Hz), 3.94 (1H, t, *J* = 3.1 Hz), 3.82 (1H, dd, *J* = 12.2, 3.4 Hz), 3.57 (2H, t, *J* = 6.7 Hz), 3.53 (2H, t, *J* = 5.2 Hz), 3.27 (2H, t, *J* = 6.7 Hz), 2.33 (1H, ddd, *J* = 13.2, 6.2, 4.0 Hz), 2.12 (1H, dt, *J* = 13.2, 6.4 Hz), 1.92 (2H, quin, *J* = 6.4 Hz), 0.97 (9H, s), 0.91 (9H, s,), 0.18 (3H, s), 0.16 (3H, s), 0.11 (6H, s). ^13^C-NMR (125 MHz, CDCl_3_) δ (ppm) 158.99, 152.94, 152.67, 146.79, 143.04, 127.61, 124.38, 122.87, 115.26, 108.27, 107.74, 87.71, 85.94, 70.82, 69.45, 62.31, 42.74, 41.88, 39.39, 37.45, 33.21, 26.04, 25.71, 18.48, 17.93, −4.58, −4.90, −5.45, −5.52. IR (cm^−1^): 2931, 1670, 1558, 1499. HR ESI-MS (*m*/*z*): Calcd. for [C_33_H_55_ClN_5_O_7_Si_2_]^+^: 724.3323 ([M+H]^+^), found 724.3340.

*1-(2-((3-((2R,4S,5R)-4-((tert-butyldimethylsilyl)oxy)-5-(((tert-butyldimethylsilyl)oxy)methyl)tetrahydro-furan-2-yl)-2-oxo-2,10-dihydro-3H-benzo[b]pyrimido[4,5-e][1,4]oxazin-9-yl)oxy)ethyl)-3-(4-chloro-butyl)urea*. (C4-8-ThioG-grasp, **2c**): **2c** was synthesized using G-clamp unit **4** and 4-chlorobutylamine [19] in 82% as a light yellow foam. ^1^H-NMR (400 MHz, CD_3_OD) δ (ppm) 7.62 (1H, s), 6.81 (1H, t, *J* = 8.6 Hz), 6.59 (1H, d, *J* = 8.6 Hz), 6.24 (1H, t, *J* = 8.2 Hz), 6.16 (1H, t, *J* = 6.1 Hz), 4.46 (1H, br), 4.03 (2H, t, *J* = 5.2 Hz), 3.94 (2H, dd, *J* = 11.6, 2.4 Hz), 3.82 (1H, d, *J* = 9.5 Hz), 3.61 (2H, t, *J* = 6.4 Hz) 3.53 (2H, t, *J* = 6.4 Hz), 3.12 (2H, t, *J* = 6.7 Hz), 2.35–2.29 (1H, m), 2.11 (1H, dt, *J* = 13.1, 6.4 Hz), 1.88–1.73 (4H, m), 0.97 (9H, s), 0.91 (9H, s,), 0.18 (3H, s), 0.16 (3H, s), 0.11 (6H, s). ^13^C-NMR (125 MHz, CD_3_OD) δ (ppm) 161.16, 156.45, 155.65, 148.10, 144.31, 129.56, 125.02, 123.52, 109.17, 108.64, 89.33, 87.51, 72.88, 70.17, 63.73, 45.49, 42.80, 40.43, 40.32, 31.10, 28.78, 26.60, 26.25, 19.37, 18.86, −4.45, −4.65, −5.25, −5.30. IR (cm^−1^): 2953, 1670, 1558. HR ESI-MS (*m*/*z*): calcd. for [C_34_H_57_ClN_5_O_7_Si_2_]^+^, 738.3480 ([M+H]^+^); found 738.3452.

(*2-((3-((2R,4S,5R)-4-((tert-Butyldimethylsilyl)oxy)-5-(((tert-butyldimethylsilyl)oxy)methyl)tetrahydro-furan-2-yl)-2-oxo-2,10-dihydro-3H-benzo[b]pyrimido[4,5-e][1,4]oxazin-9-yl)oxy)ethyl)-3-chloropropyl carbamate*. (C3 (O)-8-ThioG-grasp, **3**): **3** was synthesized using G-clamp unit **4** and 3-chloropropanol in 82% as a light yellow foam. ^1^H-NMR (400 MHz, CD_3_OD)   (ppm) 7.64 (1H, s), 6.83 (1H, t, *J* = 8.2 Hz), 6.61 (1H, dd, *J* = 8.2, 0.9 Hz), 6.37 (1H, dd, *J* = 8.2, 0.9 Hz), 6.18 (1H, t, *J* = 6.1 Hz), 4.47 (1H, dt, *J* = 5.8, 4.0 Hz), 4.19 (2H, t, *J* = 6.1 Hz), 4.06 (1H, t, *J* = 5.2 Hz), 3.96 (1H, t, *J* = 2.75 Hz), 3.94 (1H, t, *J* = 3.1 Hz), 3.83 (1H, dd, *J* = 12.2, 3.4 Hz), 3.63 (2H, t, *J* = 6.7 Hz), 3.52 (2H, t, *J* = 5.2 Hz), 3.27 (2H, t, *J* = 6.7 Hz), 2.34 (1H, ddd, *J* = 13.4, 6.1, 4.3 Hz), 2.12 (1H, dt, *J* = 13.4, 6.1 Hz), 2.06 (2H, quin, *J* = 6.1 Hz), 0.97 (9H, s), 0.92 (9H, s,), 0.18 (3H, s), 0.17 (3H, s), 0.11 (6H, s). ^13^C-NMR (125 MHz, CDCl_3_) δ (ppm) 156.62, 152.69, 152.02, 146.63, 143.16, 127.26, 124.14, 122.85, 115.58, 108.41, 107.15, 87.63, 86.02, 70.71, 68.38, 62.26, 41.89, 41.38, 40.39, 32.05, 26.04, 25.72, 18.48, 17.94, −4.56, −4.90, −5.46, −5.52. IR (cm^−1^): 2953, 1679, 1556, 1474. HR ESI-MS (*m*/*z*): calcd. for [C_33_H_54_ClN_4_O_8_Si_2_]^+^, 725.3163 ([M+H]^+^); found 725.3142.

#### 3.2.2. Synthesis of 8-ThioG Adduct **7b**

Et_3_N (100 µL, 0.72 mmol) was added to a solution of **2b** (11 mg, 0.015 mmol) and tri-Ac-8-thioG (14 mg, 0.031 mmol) in anhydrous CH_3_CN (1 mL) under an argon atmosphere. The reaction mixture was stirred at 50 °C for 12 h, and evaporated *in vacuo*. The resulting residue was purified by silica gel column chromatography (CHCl_3_/MeOH =70:1) to give **5b** as a colorless oil (9 mg, 50%). ^1^H-NMR (400 MHz, CD_3_OD) δ (ppm) 7.51 (1H, s), 6.70 (1H, t, *J* = 8.2 Hz), 6.41 (1H, d, *J* = 8.6 Hz), 6.18 (1H, t, *J* = 4.9 Hz), 6.15 (1H, d, *J* = 6.1 Hz), 6.04–6.03 (1H, m), 5.84 (1H, d, *J* = 4.6 Hz), 5.77 (1H, t, *J* = 6.1 Hz), 4.51 (1H, dd, *J* = 7.6, 2.4 Hz), 4.41–4.31 (3H, m), 3.89–3.85 (4H, m), 3.76 (1H, d, *J* = 9.5 Hz), 3.60 (2H, br), 3.29–3.21 (4H, m), 2.29 (1H, ddd, *J* = 13.0, 6.5 Hz), 2.13–2.06 (1H, m), 1.96 (2H, br), 0.93 (9H, s), 0.91 (9H, s,), 0.14 (3H, s), 0.13 (3H, s), 0.09 (6H, s) ^13^C-NMR (125 MHz, CD_3_OD) δ (ppm) 172.40, 171.44, 171.20, 161.36, 160.17, 156.20, 156.01, 155.09, 155.04, 148.62, 147.36, 144.32, 129.27, 125.38, 123.73, 117.51, 115.86, 107.64, 88.03, 87.47, 81.00, 72.63, 71.79, 64.20, 42.70, 40.58, 39.76, 31.34, 29.52, 26.66, 26.34, 20.42, 19.37, 18.88, −4.29, −4.54, −5.14. IR (cm^−1^): 2928, 1749, 1684. HR ESI-MS (*m*/*z*): calcd. for [C_49_H_73_N_10_O_15_SSi_2_]^+^, 1129.4511 ([M+H]^+^); found 1129.4469.

#### 3.2.3. N-(3-Chloropropyl)-2-(pyren-1-yl)acetamide (**6**)

3-Chloropropylamine hydrochloride (12 mg, 0.093 mmol), 1-(3-dimethylaminopropyl)-3-ethyl-carbodiimide hydrochloride (EDC, 17 mg, 0.089 mmol), 4,4-dimethylaminopyridine (DMAP, 1 mg 0.008 mmol) and Et_3_N (50 µL, 0.359 mmol) were added to a solution of 1-pyreneacetic acid (20 mg, 0.077 mmol) in anhydrous CH_2_Cl_2_ (1 mL) under an argon atmosphere. The reaction mixture was stirred at room temperature for 7 h, quenched with aqueous saturated NH_4_Cl solution, and extracted with AcOEt. The organic layer was washed with brine, dried over Na_2_SO_4_ and evaporated *in vacuo*. The resulting residue was purified by silica gel column chromatography (CH_2_Cl_2_) to give **6** as a pale yellow solid (13 mg, 50%). ^1^H-NMR (400 MHz, CDCl_3_) δ (ppm) 8.20 (2H, d, *J* = 7.6 Hz), 8.15 (3H, d, *J* = 7.9 Hz), 8.08 (1H, d, *J* = 9.2 Hz), 8.04 (1H, d, *J* = 9.2 Hz), 8.02 (1H, t, *J* = 7.9 Hz), 7.88 (1H, d, *J* = 7.9 Hz), 4.28 (2H, s), 3.31 (2H, t, *J* = 6.4 Hz), 3.25 (2H, q, *J* = 6.4 Hz), 1.78 (2H, quin, J = 6.4 Hz). ^13^C-NMR (125 MHz, CDCl_3_) δ (ppm) 171.23, 131.28, 131.16, 130.76, 129.53, 128.50, 128.16, 127.69, 127.27, 126.28, 125.60, 125.48, 125.20, 125.12, 124.62, 122.76, 42.22, 42.08, 37.28, 31.86. IR (cm^−1^): 3293, 1646, 1544. HR ESI-MS (*m*/*z*): calcd. for [C_21_H_19_ClNO]+, 336.1150 ([M+H]^+^); found 336.1161.

#### 3.2.4. Determination of the Structure of the Byproduct **8**

To a solution of **2a** (70 mg, 0.099 mmol) in MeOH (4 mL) was added NaHCO_3_ (90 mg, 1.07 mmol) under an argon atmosphere. The reaction mixture was refluxed for 25 h, filtered, and evaporated *in vacuo*. The resulting residue was purified by silica gel column chromatography (CHCl_3_/Acetone =100:0 to 50:50) to give **8** as light yellow foams (26 mg, 39%). ^1^H-NMR (400 MHz, CD_3_OD) δ (ppm) 7.60 (1H, s), 6.81 (1H, t, *J* = 8.2 Hz), 6.58 (1H, d, *J* = 8.2 Hz), 6.35 (1H, d, *J* = 8.2 Hz), 6.17 (1H, t, *J* = 6.4 Hz), 4.47 (1H, dt, *J* = 6.1, 3.5 Hz), 4.35 (2H, t, *J* = 8.2 Hz), 4.07 (2H, *J* = 4.9 Hz), 3.96–3.93 (2H, m), 3.82 (1H, dd, *J* = 12.2, 3.1 Hz), 3.78 (2H, t, *J* = 8.2 Hz), 3.55 (2H, t, *J* = 5.2 Hz), 2.33 (1H, ddd, *J* = 13.3, 6.1, 4.1 Hz), 2.12 (1H, dt, *J* = 13.3, 6.3 Hz), 0.97 (9H, s), 0.91 (9H, s,), 0.18 (3H, s), 0.16 (3H, s), 0.11 (6H, s). ^13^C-NMR (125 MHz, CD_3_OD) δ (ppm) 164.20, 156.51, 155.66, 147.98, 144.31, 129.55, 124.95, 123.42, 117.14, 109.25, 108.51, 89.34, 87.51, 72.97, 69.64, 69.37, 63.77, 43.22, 42.77, 29.54, 26.60, 26.26, 19.36, 18.85, −4.46, −4.65, −5.26, −5.30. IR (cm^−1^): 2931, 1669, 1557, 1497, 1473. HR ESI-MS (*m*/*z*): calcd. for [C_32_H_52_N_5_O_7_Si_2_]^+^ , 674.3400 ([M+H]^+^); found 674.3438.

#### 3.2.5. General Procedure of Reaction Monitoring by HPLC

Reaction was initiated by the addition of Et_3_N (20 mM) to a solution of 8-thioG-grasp derivative (0.4 mM) and triAc-8-thioG) (0.4 mM) in CH_3_CN at 50 °C. The reaction progress was monitored by HPLC at 0.5, 1, 3 and 5 h. Product yield with time course of reaction were obtained from reverse-phase HPLC analysis. (Column: Xbridge C8 3.5 μm, 3.0 × 100 mm; Solvent: A: 0.1 M TEAA buffer at pH 7.0, B: CH_3_CN, A/B= 20: 80; Flow rate: 0.5 mL/min; monitored by UV detector at 254 nm).

## 4. Conclusions

In this study, we designed new recognition molecules to covalently capture 8-thioguanosine based on the G-clamp skeleton by introducing chloroalkyl urea linker. 8-ThioG-grasp **2b** and **2c** with chloropropyl urea and chlorobutyl linker exhibited the most efficient reactivity for tri-Ac-8-thioG. It has been shown from the comparison with control compounds that the multiple hydrogen-bonded complexes contribute to the efficient reactivity. There is increasing interest in 8-thioguanosine derivatives for their biological roles in signal transduction pathways [7], the 8-thioG-grasp derivatives are expected to be a potential platform to develop specific molecules. For example, an 8-thioG-grasp derivative with a phosphate binding unit are expected to covalently trap phosphate derivatives of 8-thioguanosine and interfere their biological functions. Systematic studies are now ongoing in this line in our group, which will be reported in due course.

## Supplementary Materials

Supplementary materials can be accessed at: http://www.mdpi.com/1420-3049/20/01/1078/s1.
