# Supplementary material for: Development of New 1,3-Diazaphenoxazine Derivatives (ThioG-Grasp) to Covalently Capture 8-Thioguanosine
Source: Molecules. 2015 Jan 9;20(1):1078–87. doi: 10.3390/molecules20011078 (PMC6272678; doi:10.3390/molecules20011078)

# Supplementary Materials

**2a** $^{1}$H-NMR

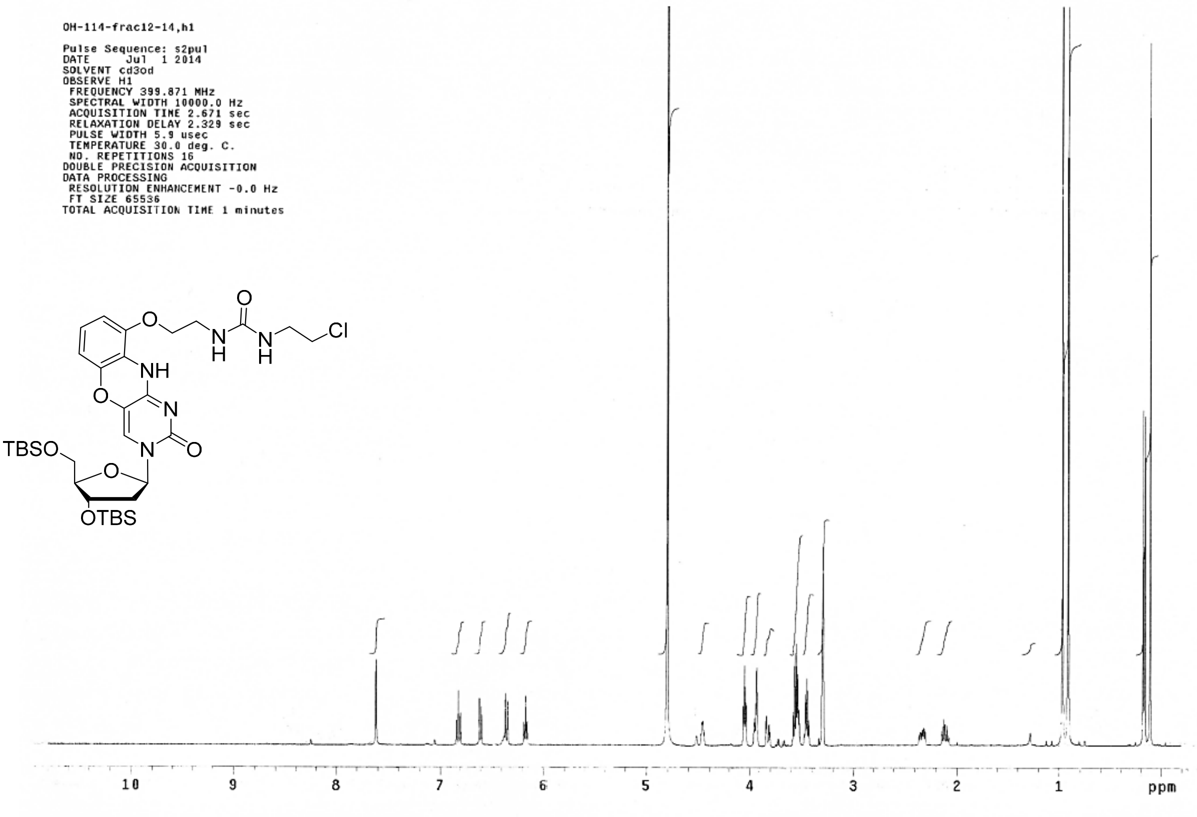


**2a** $^{13}$C-NMR

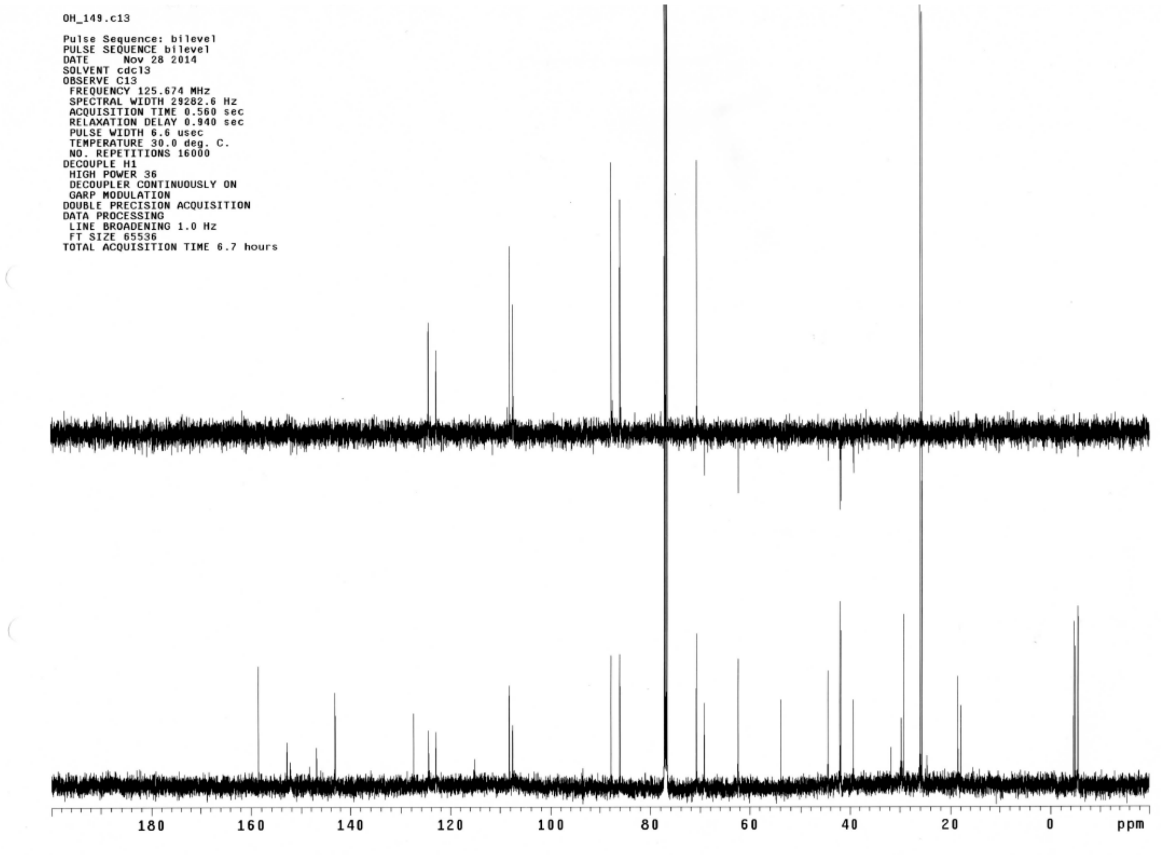

**2b** $^{1}$H-NMR

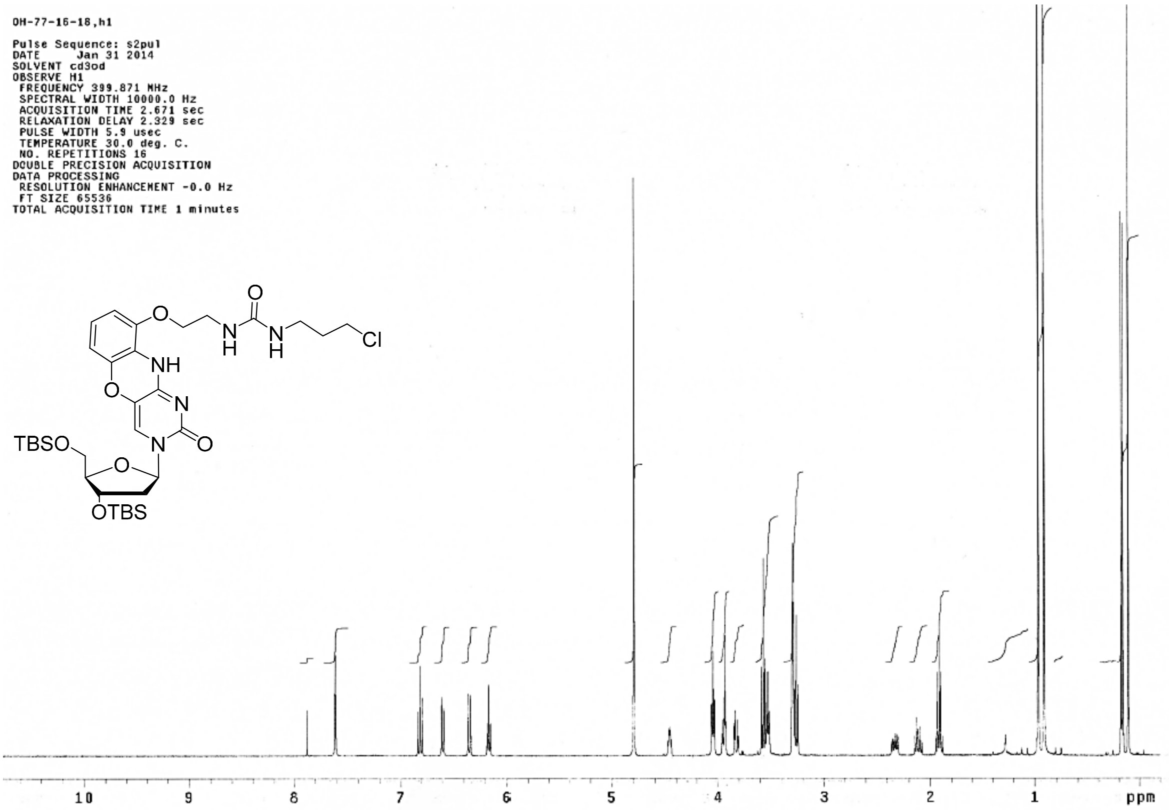


**2b** $^{13}$C-NMR

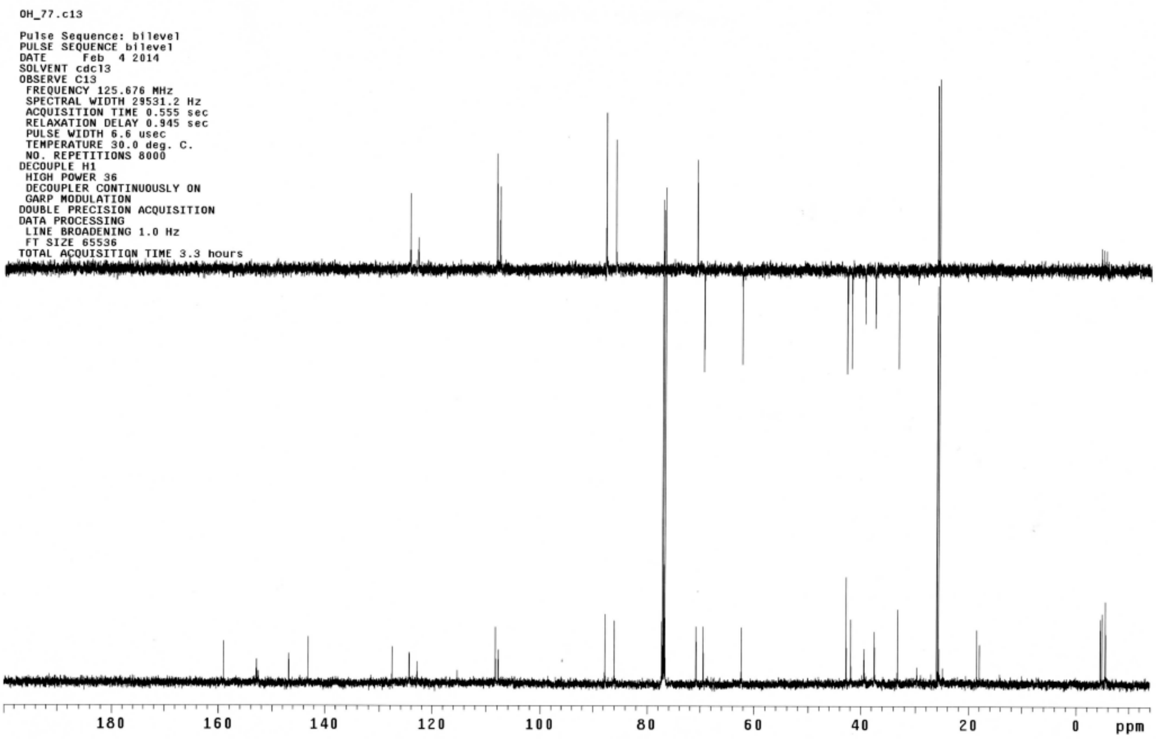

**2c** $^{1}$H-NMR

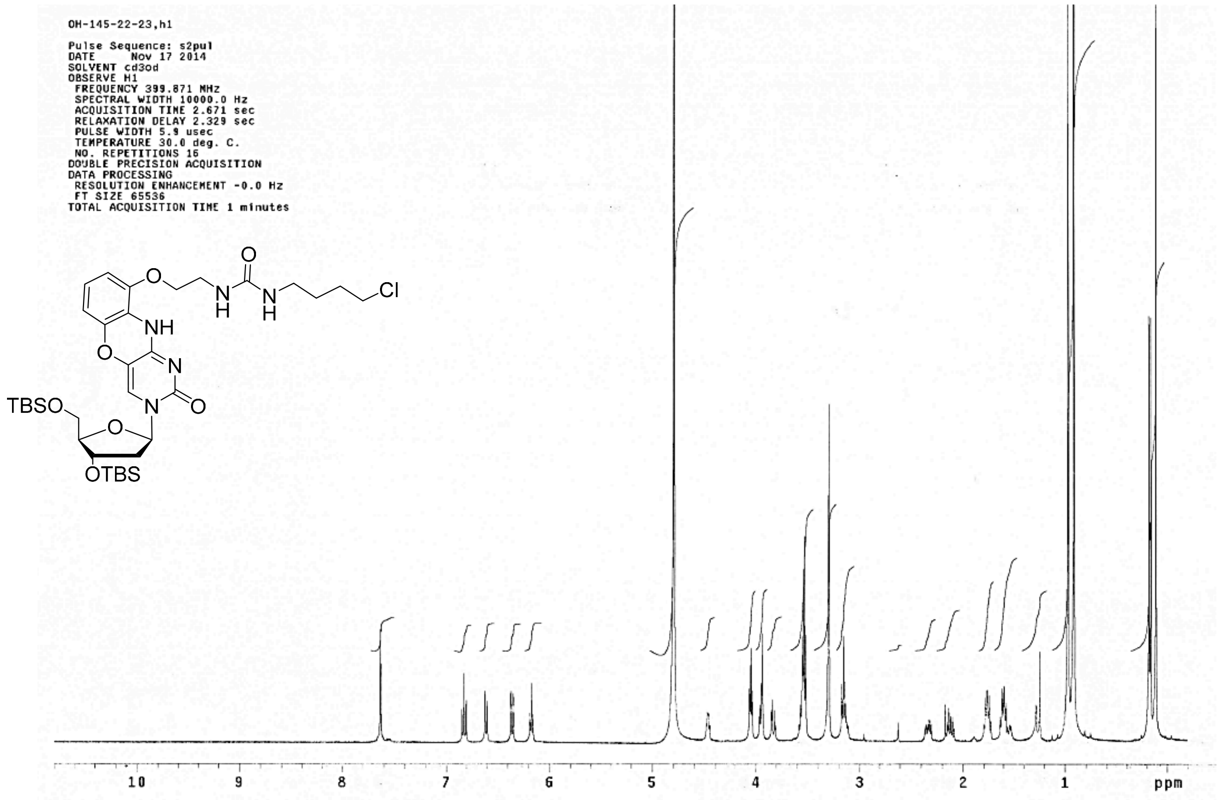


**2c** $^{13}$C-NMR

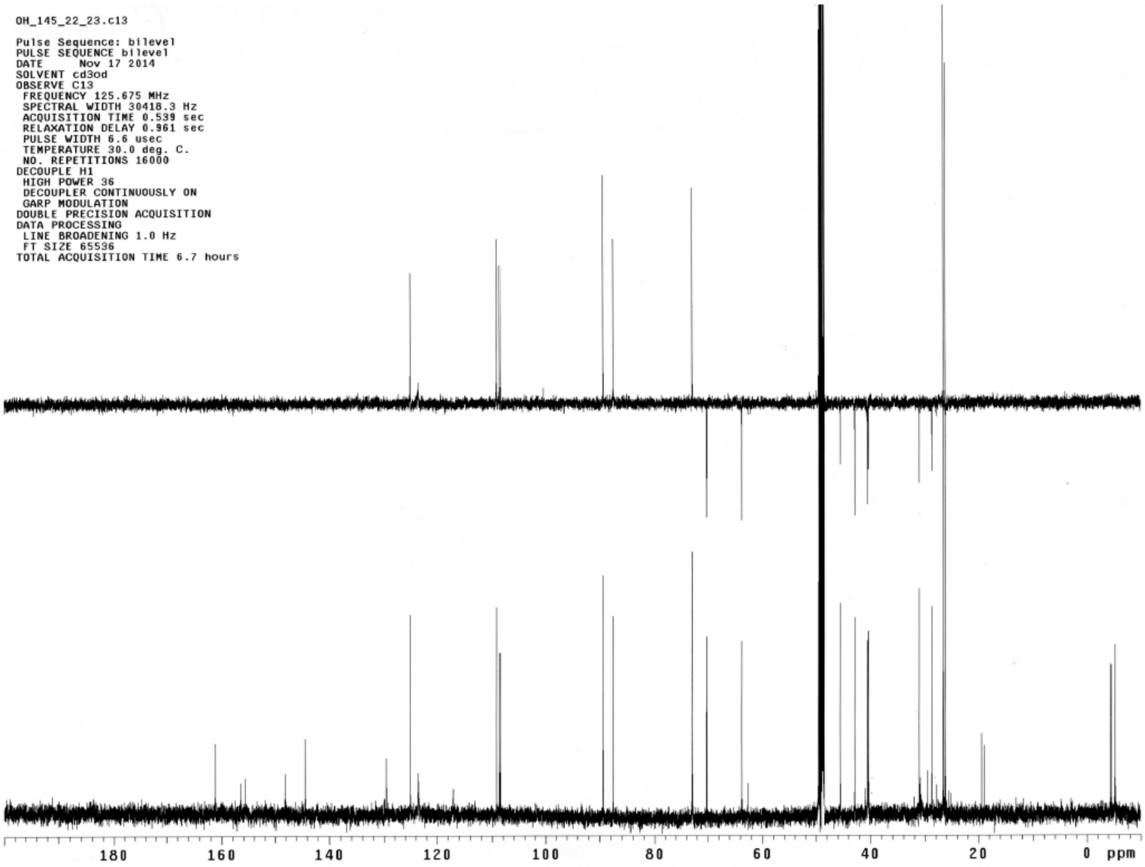

**3** $^{1}$H-NMR

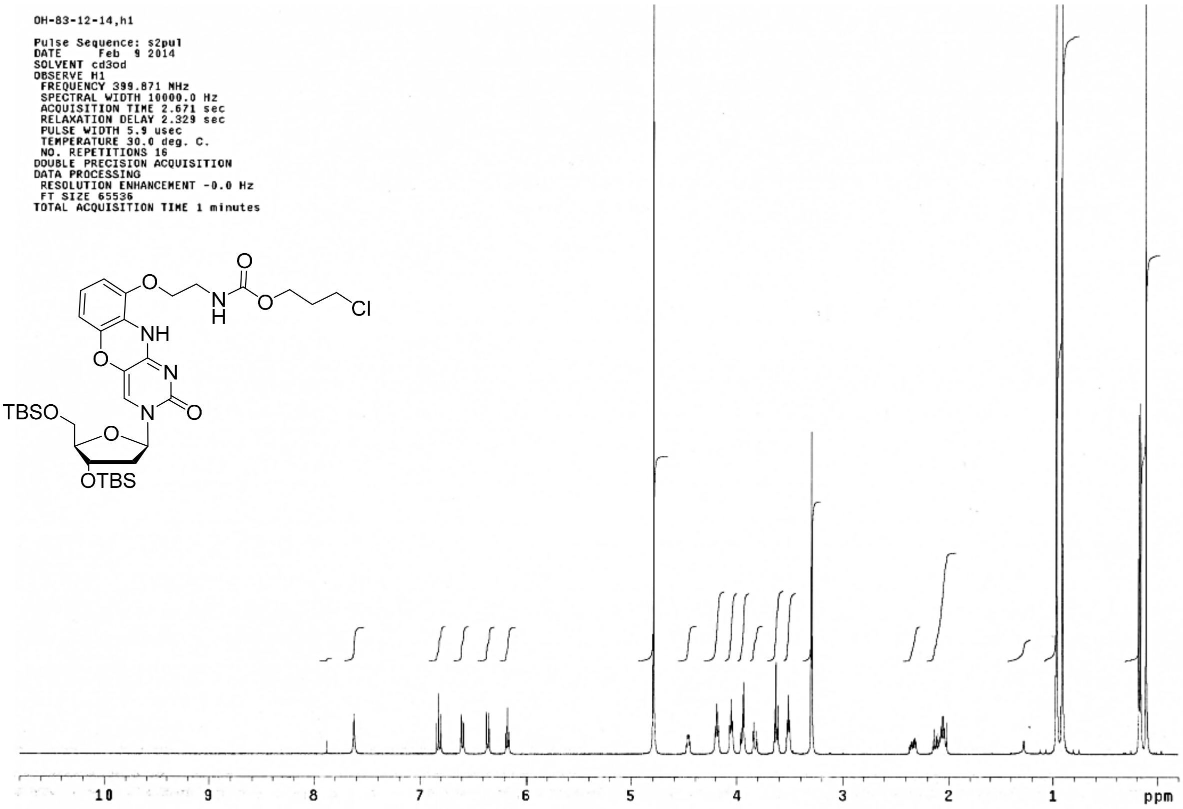


**3** $^{13}$C-NMR

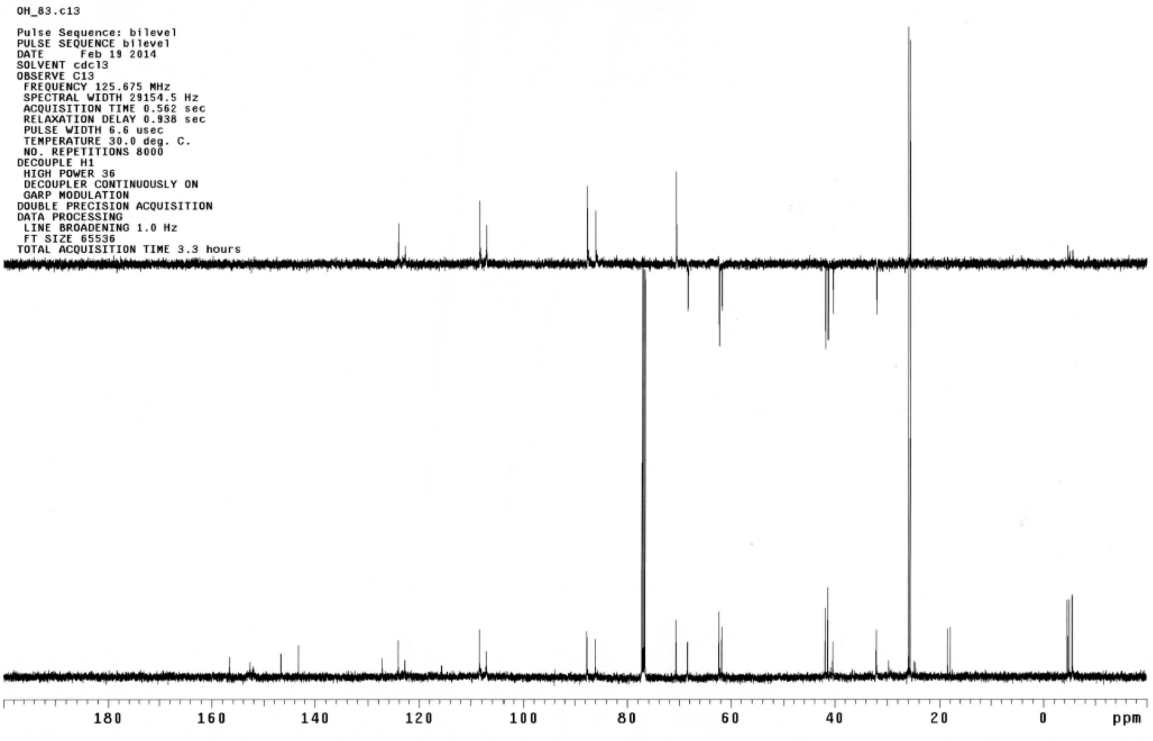

**7b** $^{1}$H-NMR

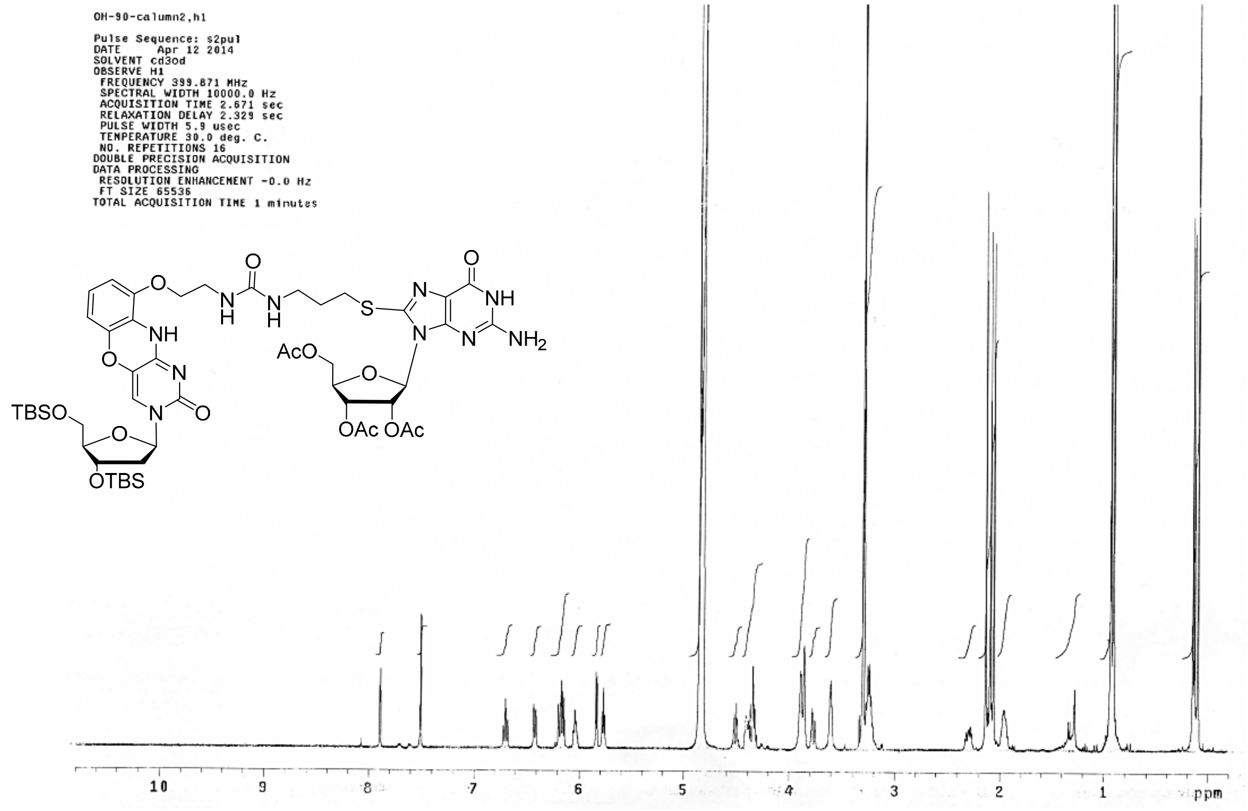


**7b** $^{13}$C-NMR

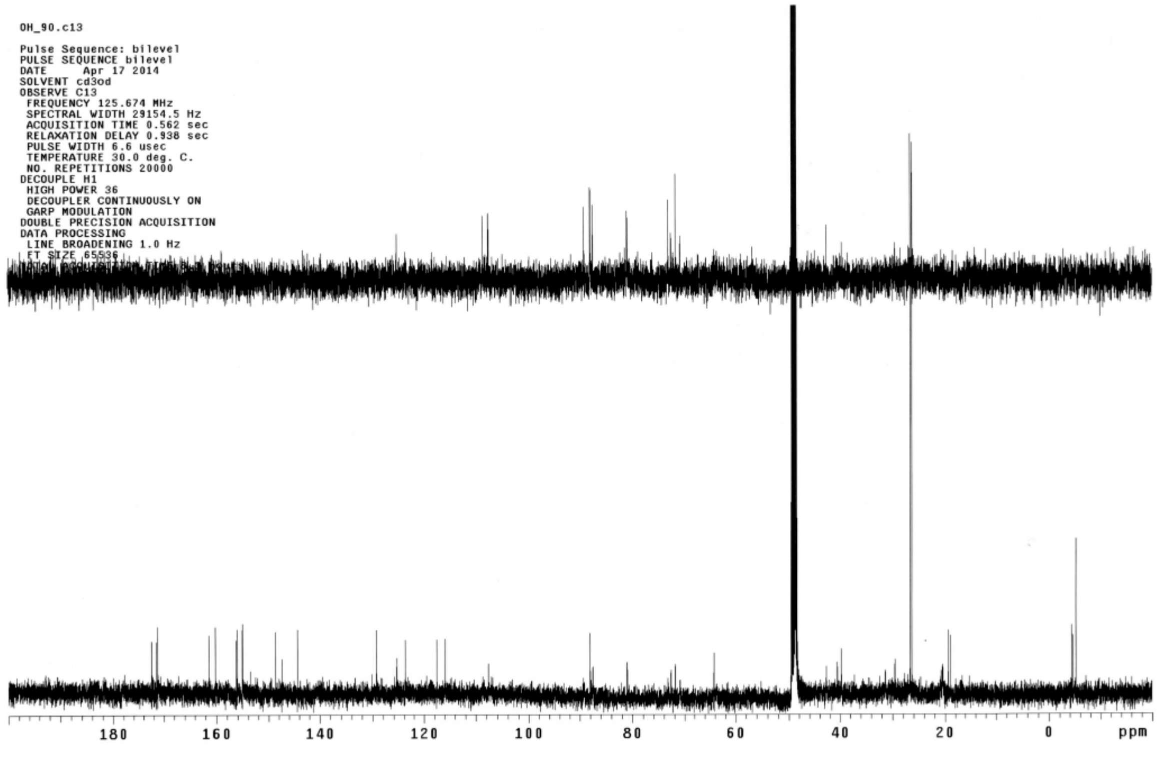

**6** $^{1}$H-NMR

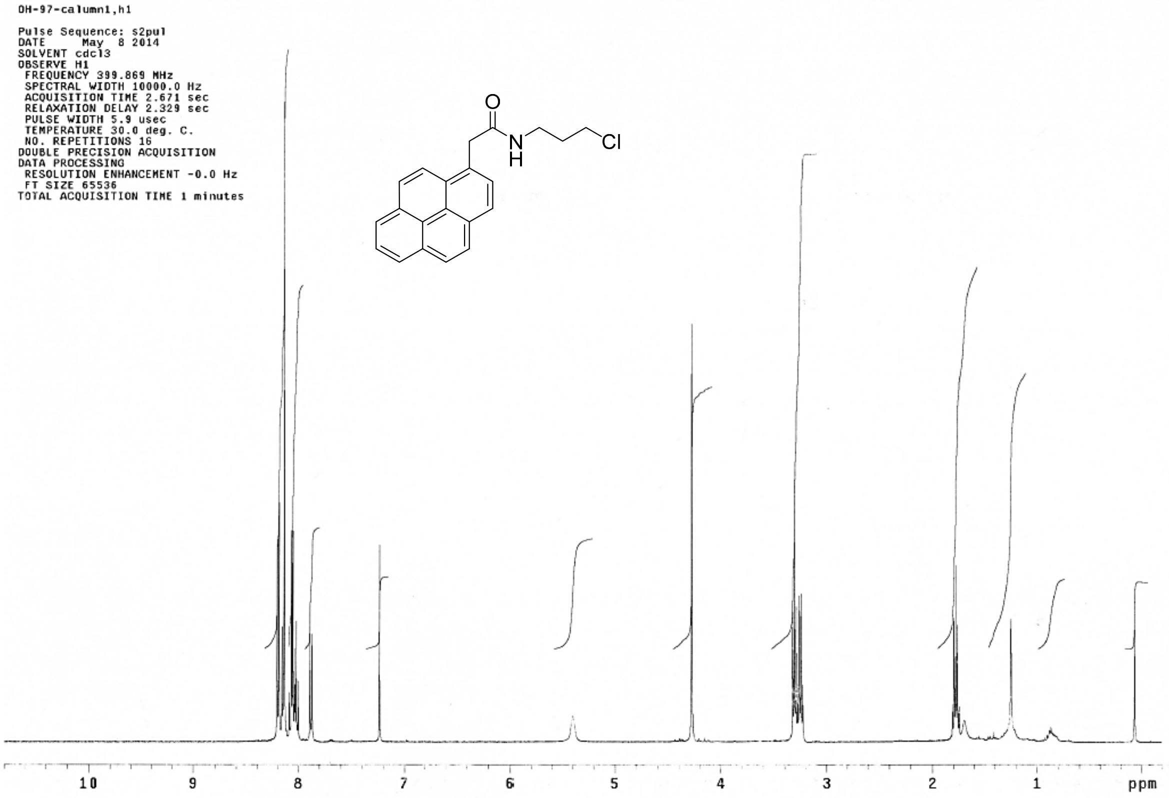


**6** $^{13}$C-NMR

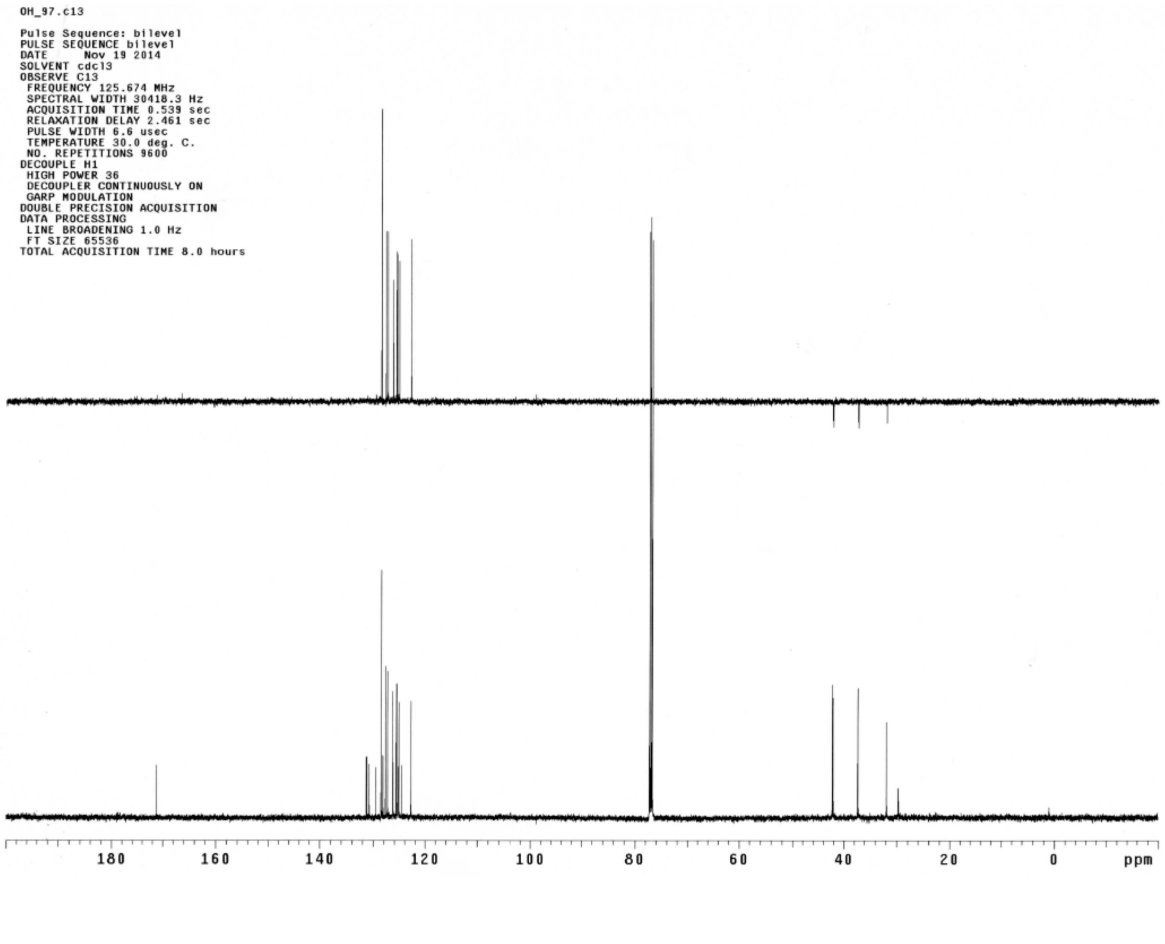

**8** $^{1}$H-NMR

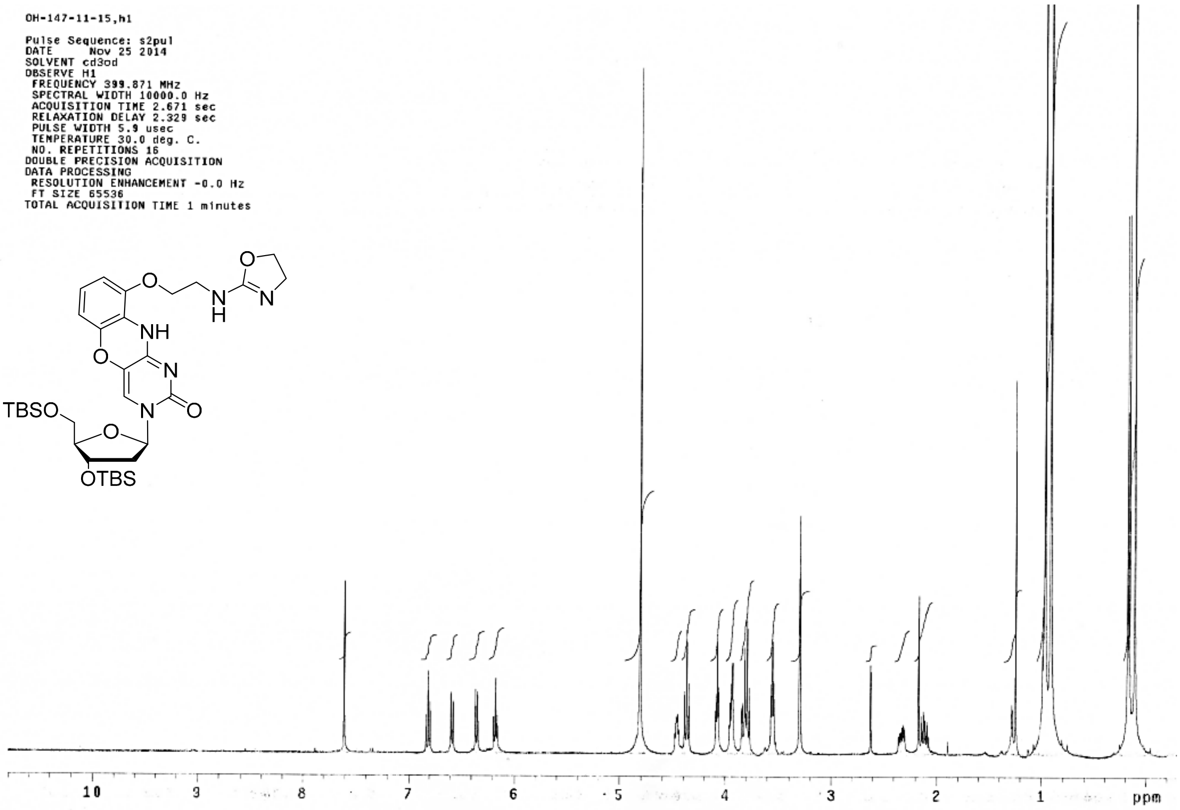


**8** $^{13}$C-NMR

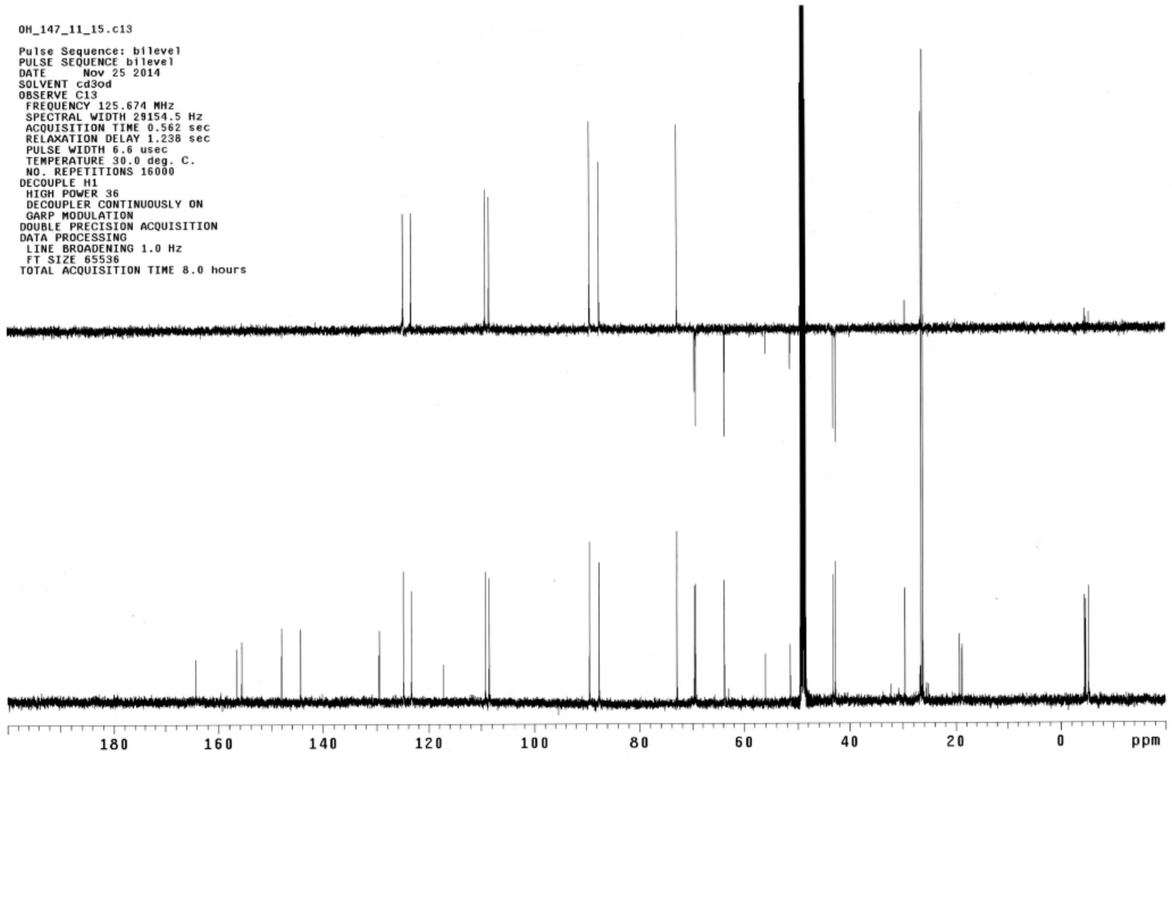

**7b** 2D-HMBC

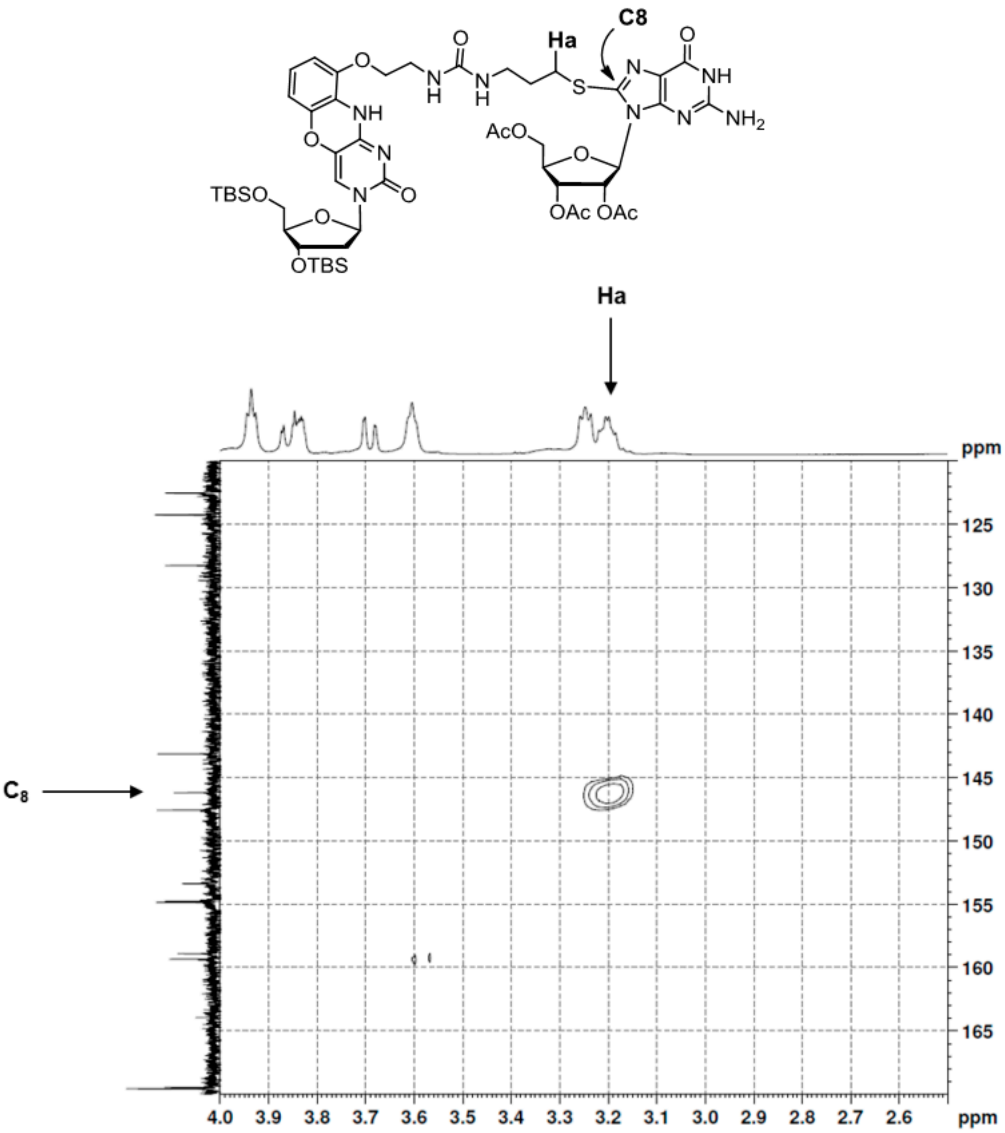
C8
Ha
TBSO
OTBS
AcO
OAc OAc
NH
NH$_2$
Ha
$C_8$
ppm
125
130
135
140
145
150
155
160
165
4.0 3.9 3.8 3.7 3.6 3.5 3.4 3.3 3.2 3.1 3.0 2.9 2.8 2.7 2.6
ppm

**8** 2D-HMBC

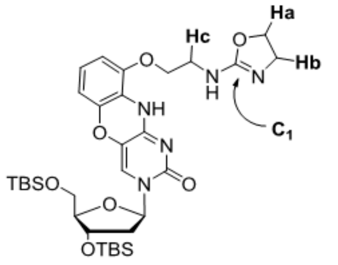
Ha
Hc
Hb
NH
N
H
C1
N
O
TBSO
OTBS

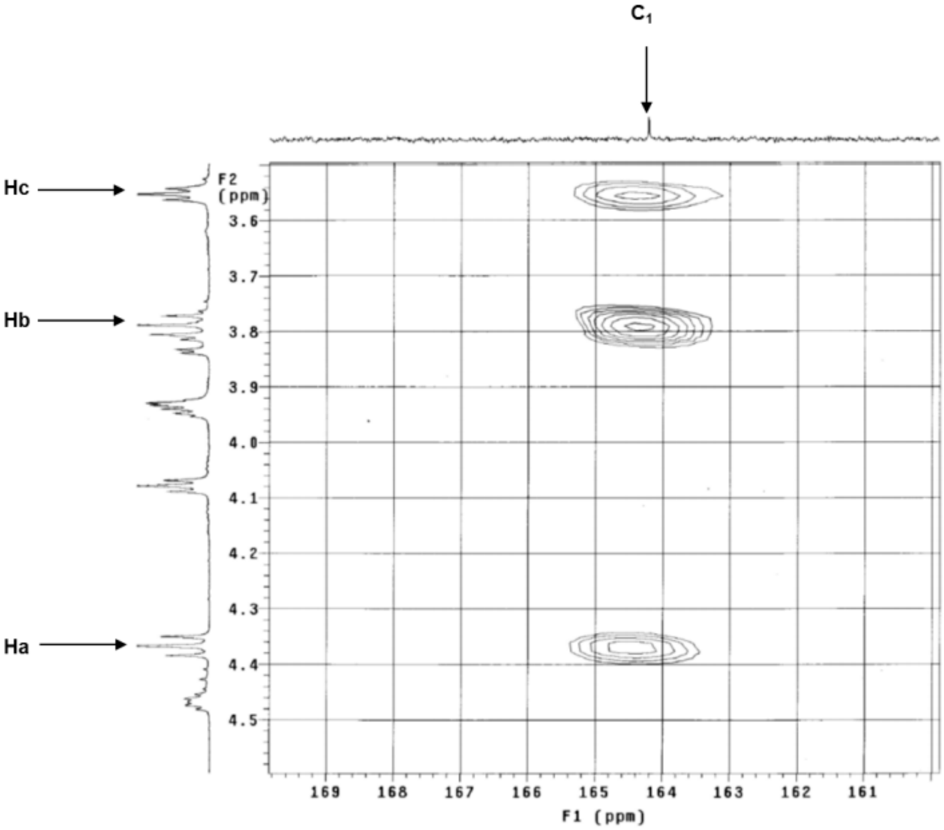
C1
Hc
Hb
Ha
F2 (ppm)
3.6
3.7
3.8
3.9
4.0
4.1
4.2
4.3
4.4
4.5
169
168
167
166
165
164
163
162
161
F1 (ppm)

## p$K_a$ Determination of 8-thioguanosine

A quartz cell containing a solution of 8-thioguanosine (20 µM) in 4 M NaOH (pH 12) was placed in a UV spectrometer and the spectrum was measured at 25 °C. A small portion of 2 M HCl was added to a quartz cell and pH was measured, and the UV spectrum was measured. This procedure was repeated. UV absorbance at 280 nm was plotted against pH and the p$K_a$ value was determined by curve-fitting method.

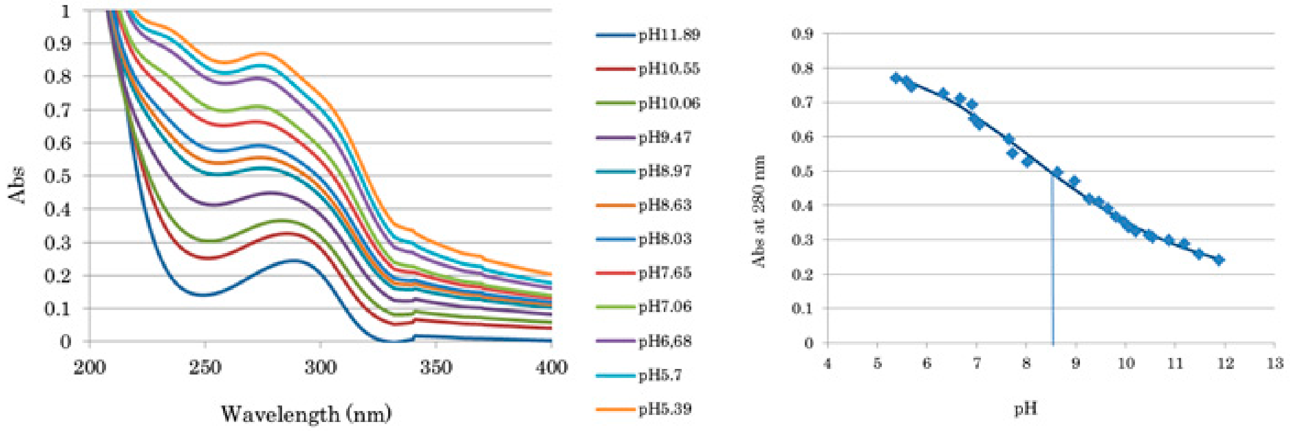

Supplement: Supplementary file 1 [file molecules-20-01078-s001.pdf]
